# Median rhomboid glossitis-developmental or candidal?

**DOI:** 10.11604/pamj.2015.21.221.5733

**Published:** 2015-07-30

**Authors:** Prashanth Panta, Sridhar Reddy Erugula

**Affiliations:** 1 Department of oral medicine and radiology, MNR Dental College and Hospital, Narsapur road, Sangareddy (502294) Telangana, India; 2 Department of Oral pathology, MNR Dental College and Hospital Narsapur road, Sangareddy (502294) Telangana, India

**Keywords:** Developmental lesion, depapillation, candidiasis

## Image in medicine

A male patient aged 20 years, visited us for routine oral examination. Incidentally, a well-demarcated reddish area of depapillation, of size 6X3 cm, was found on the dorsum of tongue near the midline. It was located 1 cm anterior to the circumvallate papillae and was roughly diamond shaped. Its surface was smooth, raised and fissured; the opposing palatal mucosa was non-erythematous (kissing lesion was absent). History revealed that it was stable since childhood and was occasionally associated with burning sensation. The patient was otherwise healthy, with no history of tobacco usage or diabetes. Based on history and clinical examination, a diagnosis of “median rhomboid glossitis” was arrived. The patient was given fluconazole (50mg, 1 time/day for 14 days) to rule out candidiasis, but, the lesion did not regress. Median rhomboid glossitis is also known as “central papillary atrophy of the tongue”. It is a benign lesion that shows a marked male predilection and occurs in less than 1% of adult population. Initially, it was believed to be developmental in origin, but, during the recent years, it has been considered as a variant of candidiasis. The differential diagnosis includes erythroplakia, geographic tongue, granular cell tumor. Patients with this condition should be reassured about its harmless nature and no treatment is required for asymptomatic cases.

**Figure 1:**
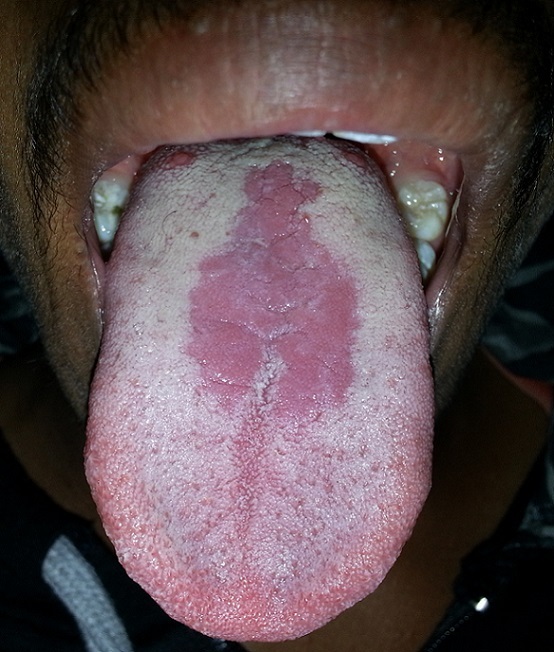
Central papillary atrophy

